# Metabolic Profiling in Rheumatoid Arthritis, Psoriatic Arthritis, and Psoriasis: Elucidating Pathogenesis, Improving Diagnosis, and Monitoring Disease Activity

**DOI:** 10.3390/jpm12060924

**Published:** 2022-06-02

**Authors:** Erika Dorochow, Michaela Köhm, Lisa Hahnefeld, Robert Gurke

**Affiliations:** 1 Pharmazentrum Frankfurt/ZAFES, Institute of Clinical Pharmacology, University Hospital of Goethe-University, Theodor-Stern-Kai 7, 60590 Frankfurt am Main, Germany; dorochow@med.uni-frankfurt.de (E.D.); hahnefeld@med.uni-frankfurt.de (L.H.); 2 Fraunhofer Institute for Translational Medicine and Pharmacology (ITMP), Theodor-Stern-Kai 7, 60596 Frankfurt am Main, Germany; michaela.koehm@itmp.fraunhofer.de; 3 Fraunhofer Cluster of Excellence for Immune-Mediated Diseases (CIMD), Theodor-Stern-Kai 7, 60596 Frankfurt am Main, Germany; 4 Department of Rheumatology, University Hospital Frankfurt, Theodor-Stern-Kai 7, 60596 Frankfurt am Main, Germany

**Keywords:** biomarkers, precision medicine, metabolomics, lipidomics, psoriasis, arthritis, rheumatoid, arthritis, psoriatic, immune-mediated inflammatory diseases

## Abstract

Immune-mediated inflammatory diseases (IMIDs), such as rheumatoid arthritis (RA), psoriatic arthritis (PsA), and psoriasis (Ps), represent autoinflammatory and autoimmune disorders, as well as conditions that have an overlap of both categories. Understanding the underlying pathogeneses, making diagnoses, and choosing individualized treatments remain challenging due to heterogeneous disease phenotypes and the lack of reliable biomarkers that drive the treatment choice. In this review, we provide an overview of the low-molecular-weight metabolites that might be employed as biomarkers for various applications, e.g., early diagnosis, disease activity monitoring, and treatment-response prediction, in RA, PsA, and Ps. The literature was evaluated, and putative biomarkers in different matrices were identified, categorized, and summarized. While some of these candidate biomarkers appeared to be disease-specific, others were shared across multiple IMIDs, indicating common underlying disease mechanisms. However, there is still a long way to go for their application in a routine clinical setting. We propose that studies integrating omics analyses of large patient cohorts from different IMIDs should be performed to further elucidate their pathomechanisms and treatment options. This could lead to the identification and validation of biomarkers that might be applied in the context of precision medicine to improve the clinical outcomes of these IMID patients.

## 1. Introduction

### 1.1. Immune-Mediated Inflammatory Diseases

**Figure 1:**
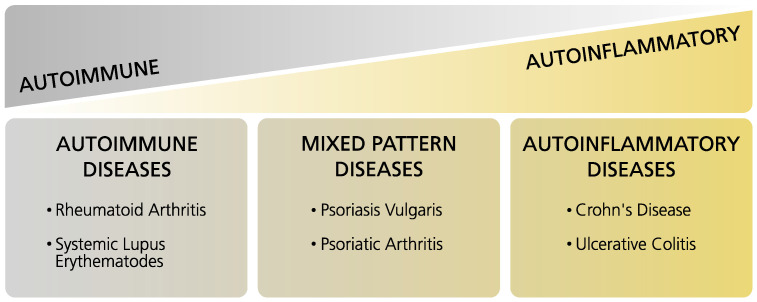
Tabular overview of immune-mediated inflammatory diseases (IMIDs). Adapted from Reference [3].

Immune-mediated inflammatory diseases (IMIDs) encompass conditions of the “inflammatory spectrum” in which autoinflammatory and autoimmune disorders represent the two ends of the spectrum, and, within this field, a multitude of conditions with overlapping characteristics can be found [1]. Moreover, autoinflammatory and autoimmune diseases have different etiologies: On the one hand, autoinflammatory diseases are characterized by systemic inflammation that is caused by the innate immune system. On the other hand, autoimmune diseases show inflammation and degradation of local tissue with the involvement of the adaptive immune system and subsequent high levels of autoantibodies and self-reactive lymphocytes [2]. Rheumatoid arthritis (RA), psoriatic arthritis (PsA), and psoriasis (Ps) are classified as IMIDs, whereby RA represents an autoimmune disease, and PsA and Ps are assigned to the mixed-pattern diseases (Figure 1) [3]. This review focuses on those three IMIDs due to the clinical similarities of RA and PsA, particularly in early undifferentiated disease stages, and since both diseases display poly-articular phenotypes and there is a high need for discriminative biomarkers for early diagnosis and disease management. This is also important in the case of PsA and Ps, as diagnosis is often delayed, and this can lead to worse outcomes and irreversible damage [4]. The clinical phenotypes of the aforementioned diseases differ from those of other IMIDs, e.g., systemic lupus erythematosus (SLE) or inflammatory bowel disease (IBD), in which different organ systems may be involved. Thus, the scope of this review mainly encompasses RA, PsA, and Ps. Nevertheless, comparisons to other IMIDs are occasionally made to enable a look at the wider picture. We further point out differences and similarities in the metabolic profiles of IMIDs, discuss the observed findings, and propose directions for future studies.

### 1.2. Rheumatoid Arthritis

**Figure 2:**
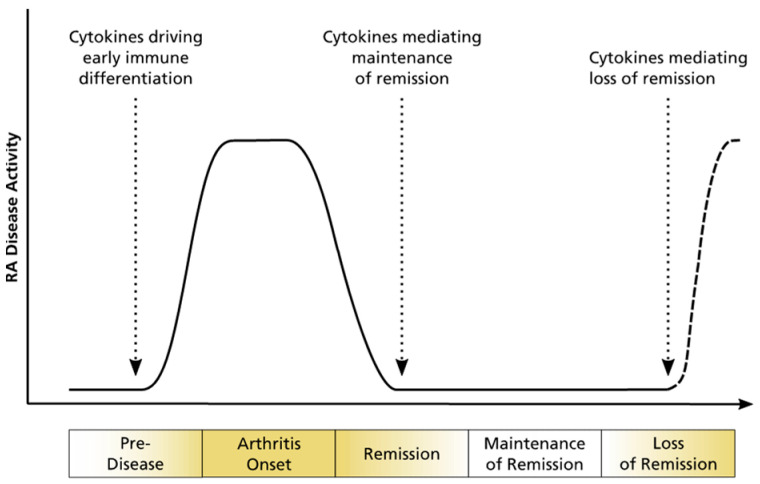
Schematic illustration of disease progression in RA with implied possible loss of remission. Adapted from Reference [13].

RA is a chronic autoimmune disease and represents the most common immune-mediated inflammatory disease [5,6]. Approximately 0.5% to 1% of the world population is affected by RA, with a higher prevalence in women than in men [5]. Its clinical picture is characterized by inflammation of the synovium, leading to mainly symmetric polyarthritis [7]. Inflammation causes changes in the cell environment, such as hypoxic conditions, and leads to the release of cytokines and chemokines, which alter the metabolic profile and determine disease progression (Figure 2) [8,9]. It can progress as an erosive disease, leading to bone erosion, with disability and movement restrictions in the involved joints. Additionally, RA patients often suffer from comorbidities, such as cardiovascular diseases. Specific diagnosis is difficult [10] since it relies on classification criteria that include clinical phenotype, imaging findings, and the combined measurements of anti-CCP antibodies (ACPAs) and rheumatoid factors (RFs) in blood. ACPAs are only detectable in 60–80% of RA patients, and RFs are nonspecific due to their additional occurrence in patients with other autoimmune diseases, as well as in healthy subjects [11]. Especially in early disease stages, symptoms are highly variable, and the distinction from other types of arthritides is limited [12].

### 1.3. Psoriatic Arthritis

PsA is a mixed-pattern disease, as it features characteristics of an autoimmune, as well as an autoinflammatory disease. It is characterized by inflammatory processes in the musculoskeletal structures which can lead to the impairment of physical functioning [4]. Enthesial musculoskeletal structures are the main target of inflammation, but the synovium is also greatly affected and clinical phenotypes may have similarities to RA. Besides bone erosions, bone proliferation is typically detected [14], which differentiates PsA from RA. Moreover, PsA patients have a mainly asymmetric form of arthritis (oligo- or polyarthritis) and are commonly affected by non-articular manifestations and comorbidities, e.g., uveitis, cardiovascular diseases, and obesity [15]. There are no known disease-specific antibodies, but it is well established that a class I major histocompatibility complex (MHC) allele, HLA-B27, is associated with PsA [16].

### 1.4. Psoriasis

Ps is a chronic immune-mediated inflammatory skin disorder that is also considered a mixed-pattern disease. Its worldwide prevalence ranges from 0.91% to 8.5% [17]. Plaque psoriasis, which is also called psoriasis vulgaris, is the most frequent form and makes up more than 80% of all psoriasis cases. Three other subtypes of psoriasis are known, including guttate psoriasis, erythrodermic psoriasis, and pustular psoriasis [18]. Only the indications plaque psoriasis, cutaneous psoriasis, or just psoriasis were considered during our search of the literature for this review and subsumed under the term psoriasis (Ps). The clinical picture of psoriasis is characterized by erythematous and scaly patches (so-called “plaques”) which are located on the head, ears, elbows, and knees, as well as gluteal and umbilical areas most of the time. Due to these skin changes and stigmatization, Ps patients frequently suffer from psychosocial problems. There is also a high rate of comorbidities, such as psoriatic arthritis, cardiovascular diseases, and obesity [18].

### 1.5. Disease Progression from Psoriasis to Psoriatic Arthritis

**Figure 3:**
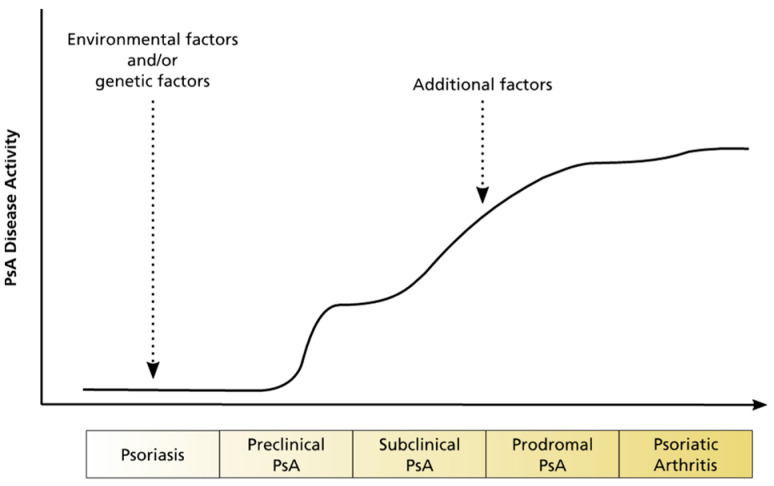
Schematic illustration of disease progression from Ps to PsA. Adapted from Reference [14].

Usually, Ps precedes the diagnosis of PsA, and approximately 30% of Ps patients will develop PsA [16]. The factors that cause PsA are complex and include individual variations of the genotype, phenotype, and the immune system, as well as environmental factors (Figure 3). In general, the time to diagnosis of active PsA is delayed when disease activity is high and musculoskeletal inflammation with radiographic damage can already be observed. This results from the lack of diagnosis criteria and well-validated diagnostic tools. Further research might improve early PsA diagnosis in Ps patients at risk for PsA and prevent disease progression in its early stages [19]. Furthermore, PsA offers the potential for personalized or precision medicine, as the majority of PsA patients does not achieve optimal treatment results [20].

### 1.6. Precision Medicine, Biomarkers, and Metabolomics

As defined by the National Institutes of Health (NIH), precision medicine represents an emerging approach for personalized disease treatment and prevention. Precision medicine aims at improving clinical outcomes by incorporating individual differences of patients, such as in genotypes and metabolic profiles, and clustering these patients into subgroups that will likely benefit from a common treatment approach. Patient clustering presumably leads to the identification of biomarkers that are distinct for specific patient subgroups [21,22].

**Figure 4:**
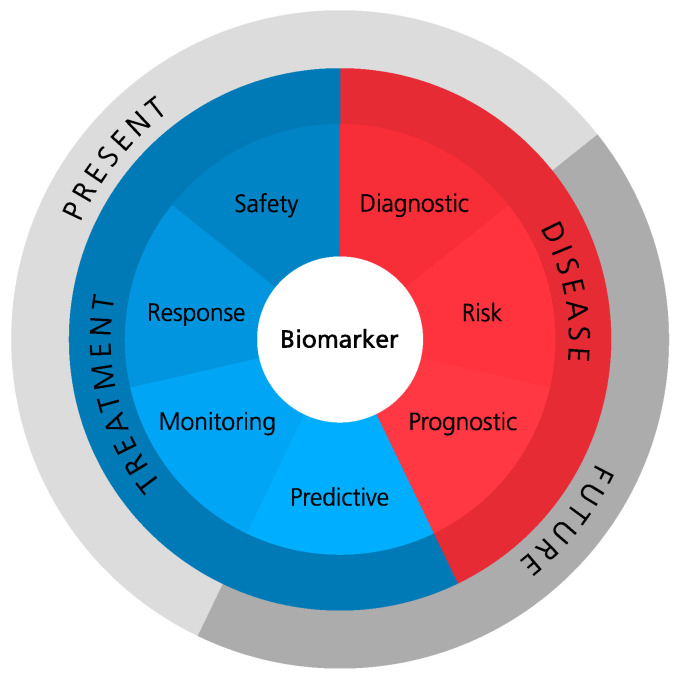
Biomarker categories according to the FDA–NIH Biomarker Working Group BEST Resource.

Biomarkers can be defined as measurable indicators of normal biological processes, pathogenic processes, or biological responses to treatment. Their utilization is very versatile as they can be employed as diagnostic tools, for monitoring disease activity and severity, for prediction of treatment outcome, as well as for the prognosis of disease progression. Seven distinct biomarker categories were defined by the FDA–NIH Biomarker Working Group: diagnostic, risk, prognostic, predictive, monitoring, response, and safety, as displayed in Figure 4 [23].

One example of indicators that can potentially act as biomarkers are low-molecular-weight metabolites which collectively constitute the metabolome of a living organism. The scientific approaches that deal with profiling the metabolome or specific metabolites are summarized under the term “metabolomics” [24,25]. Moreover, metabolomics is one branch of the “omics” sciences, which also comprise genomics, transcriptomics, and proteomics. The integration of multiple “omics” approaches is called “multi-omics” and enables further understanding of underlying disease mechanisms and, thus, might pave the way for the development of novel treatment options. Moreover, metabolite panels rather than single metabolites might be more suitable for the application as biomarkers [26,27].

In this review, we provide an overview of metabolites that might be employed as biomarkers for various applications, e.g., diagnosis, disease activity monitoring, and drug-response prediction, in RA, PsA, and Ps. A systematic review of the literature in PubMed, using the search terms provided in Supplementary Table S3, was performed. The last search inquiry was entered on 19 January 2022. References from the collected articles were also reviewed, and articles that were identified through other sources (e.g., Google Scholar search or recommendations) were additionally included. This search strategy yielded around 1100 titles that were assessed based on the following criteria: (1) study involved subjects suffering from either RA, PsA, or Ps; (2) study investigated at least one of the seven biomarker categories defined by the FDA–NIH Biomarker Working Group BEST Resource; and (3) study conducted an omics or similar experiment. In total, 67 publications were chosen for this review; they are listed in Supplementary Table S1.

## 2. Rheumatoid Arthritis

RA was addressed in numerous studies, using metabolomics analysis, and a broad range of interesting compounds was identified (Table 1). These metabolites have the potential to be employed as biomarkers in various matrices, and they mainly include amino acids, tricarboxylic acid (TCA) cycle metabolites, lactic acid, glucose, and cholesterol, as well as lipids.

In particular, lower concentrations of glutamine were reported in plasma [28] and serum [26,29,30]. Glutamine is the most abundant free amino acid in human blood and acts as an immunomodulator since it affects T-cell-mediated immunity [29]. Furthermore, it is necessary for protein biosynthesis and is connected to energy generation, as it is providing intermediates for the TCA cycle [31]. For histidine, which was linked to inflammation, as it displays anti-inflammatory and antioxidant properties [32], decreased levels were described in plasma [32,33] and serum [29,34,35]. Nonetheless, histidine supplementation was reported to be not beneficial for the treatment of RA [35]. Histidine levels also showed to be negatively correlated with different disease activity scores in serum [35] and plasma [32]; thus, it might be a useful monitoring biomarker for RA disease activity. Certain biomarkers can also be used to determine the risk of developing a specific disorder. One example of risk biomarkers is symmetric dimethylarginine (SDMA), as it showed associations with hypertension and hyperlipidemia in RA patients [36]. Changes in levels of valine, leucine, and isoleucine, which are classified as branched-chain amino acids (BCAAs), were also found. In an adjuvant-induced (AIA) rat model, valine and leucine showed lower concentrations in plasma [33]. Additionally, decreased concentrations of valine and isoleucine were found in the serum of RA patients [30,37]. The decrease in valine concentrations might result from its consumption to fuel the TCA cycle due to lower glycolytic activity [33]. By contrast, elevated BCAA levels were measured in the synovial fluid and articular joints, and they might indicate changes in inflammatory conditions, as BCAA are involved in the promotion of inflammation, migration, and oxidative stress [30,38]. The observed increase of BCAA levels in the synovial fluid might also be the result of collagen breakdown [33]. Moreover, responders to treatment with etanercept, a tumor necrosis factor inhibitor (TNFi), showed increased serum levels of BCAAs [39]. The taurine biosynthesis pathway was also found to be dysregulated in RA patients, and lower taurine levels were detected in patients with RA [28]. Additionally, taurine showed the potential to be employed as a biomarker for response to methotrexate (MTX) and TNFi therapy in RA serum samples, as elevated levels were found in MTX responders and decreased levels were found in TNFi responders [40].

Concentrations of citric acid, a TCA cycle metabolite, were reported to be lower in the blood and urine of RA patients when compared to healthy controls [29,41]. Serum levels of citric acid also appeared to be suitable to predict response to abatacept, which is a T-cell activation inhibitor, as it was elevated in responders [40]. In addition to citric acid, lactic acid showed a positive correlation with infliximab treatment in another study [42]. Lactic acid levels were higher in RA serum [26,29,43], plasma [44], and synovial fluid [45]. This might be the result of increased energy demand and production under hypoxic conditions, and it is caused by permanent inflammation. As a consequence, the enzyme lactate dehydrogenase is upregulated [26,45], which then leads to pathogenic angiogenesis, pannus formation, and the amplified catabolization of pyruvic acid, an intermediate of glucose, to lactic acid [45]. This is in accordance with the observed lower levels of pyruvic acid and glucose in the blood of RA patients [29]. In general, levels of lactic acid might be an indicator of active arthritic inflammation, as increased levels were also found in other arthritic conditions [26], and a suitable candidate for disease activity monitoring, since its concentrations were described to positively correlate with disease activity scores in the plasma [44] and serum [43] of RA patients.

Acylcarnitine levels were decreased in RA patients compared to healthy controls [46], and short-chain acylcarnitines were reported to be lower concentrated in the serum of women who later develop RA [47]. Carnitine plays a key role in fatty acid metabolism, as it is responsible for the transport of fatty acids into the mitochondrial matrix, where β-oxidation is performed to generate energy [48]. It is also known that low carnitine levels are linked to aging, chronic inflammation, chronic renal failure, malnutrition, and cachexia [47]. Thus, decreased levels of acylcarnitines might result from impaired carnitine biosynthesis [46] due to inflammation and cachexic conditions in RA patients. Multiple studies described lower total cholesterol levels in the serum and plasma of RA patients compared to healthy controls or in a cohort that is going to develop RA in the future [29,34,49,50,51,52]. This might result from chronic inflammation and the subsequent upregulation of lipid catabolism and β-oxidation. Furthermore, the development of RA might be caused by a greater susceptibility to inflammation due to a dysregulated lipid profile or the same factors, which lead to the development of RA and induce dysregulation of the lipid profile [53]. By contrast, the levels of total cholesterol were also found to be elevated in the serum [49] and plasma [44,54] of RA patients. It was suggested that lower total cholesterol levels are associated with active inflammation [34] and that the levels are adjusted to homeostasis after treatment with anti-inflammatory drugs [55]. This is in accordance with the described increases in cholesterol levels after the administration of an IL-6 receptor blocker [56], a Janus kinase (JAK) inhibitor [57], or DMARDs [55] to RA patients. Cholesterol is also associated with RA disease activity, as positive and negative correlations were reported in plasma [44] and serum [53,58], respectively. Moreover, it was reported that serum lipids, i.e., lysophosphatidylcholine, phosphatidylcholine, ether-linked phosphatidylethanolamine, and sphingomyelin subclasses, were also negatively correlated with RA disease activity. The serum lipidome might additionally determine RA disease progression and response to DMARD treatments [59].

**Table 1:** Changed metabolites in rheumatoid arthritis (RA). Biomarker category highlighted in bold: Corresponding metabolites listed in column “Results”.

Reference	Matrix	Instruments	Disease Activity and/or Severity	Patients Treated?	Biomarker Categories *		Results
Rantapää-Dahlqvist 1991 [50]	Plasma	ELISA and radioimmunoassay kit	Median	Yes	**D**	↑	lipoprotein (a)
						↓	cholesterol, HDL
Lauridsen 2010 [44]	Plasma	1H-NMR	Mixed: DAS28 active (5.0), in remission (2.6)	N/A	**D**, M	↑	cholesterol C-21, lactate, acetylated glycoprotein, unsaturated lipid
						↓	HDL
Chandrasekharan 2018 [36]	Plasma	LC–MS	DAS28 (2.7)	Yes (mixed)	**D**, Ri	↑	L-ornithine, ADMA, SDMA
						↓	L-citrulline
Sasaki 2019 [32]	Plasma	CE–Q-TOF-MS	Mixed: DAS28-ESR active (>3.2), inactive (<3.2)	No biologicals	**D**, M	↑	N,N-dimethylglycine, urea
						↓	guanidoacetic acid, histidine, homoarginine or N6,N6,N6-trimethyllysine
Kishikawa 2021 [60]	Plasma	CE–TOF-MS	N/A	N/A	**D**	↑	ethanolamine phosphate, ATP, GDP, ADP, 6-aminohexanoic acid, taurine
						↓	xanthine
Liu 2021 [33]	Plasma	UPLC–LTQ/Orbitrap- MS	N/A	No (rat model)	**D**	↑	glutamic acid, arginine, methionine
						↓	proline, valine, tyrosine, phenylalanine, leucine, glycine, tryptophan, histidine, threonine
He 2021 [28]	Plasma	GC–Q-TOF-MS	Mixed: medium, high	N/A	**D**	↓	glutamine, cysteine, citric acid
Krähenbühl 1999 [46]	Plasma, Urine	Radioenzymatic assay and HPLC	DAS (4.35)	N/A	**D**	↑	in plasma: long-chain acylcarnitine
						↓	in urine: carnitine
Madsen 2011 [34]	Serum	GC–TOF-MS and UPLC–MS	Mixed: DAS28 (4.06)	Yes (mixed)	**D**	↑	glyceric acid, D-ribofuranose, hypoxanthine
						↓	histidine, threonic acid, methionine, cholesterol, asparagine, threonine
Ouyang 2011 [29]	Serum	1H-NMR	N/A	Yes (mixed)	**D**	↑	lactic acid
						↓	glucose, creatinine, pyruvate, citrate, proteinogenic AAs, glycerides, phosphocholine
Young 2013 [43]	Serum	1H-NMR	N/A	Naive for DMARDs	**D**, M	↑	3-hydroxybutyrate, lactate, acetylglycine, taurine, glucose
						↓	LDL-CH3, LDL-CH2, alanine, methylguanidine, lipids
Jiang 2013 [26]	Serum	GC–TOF-MS and UPLC–QTOF-MS	Mixed: active, mild, or moderate	N/A	**D**, M	↑	homoserine, glyceraldehyde, lactic acid, dihydroxyfumaric acid, aspartic acid
						↓	4,8-dimethylnonanoyl carnitine
Zabek 2016 [37]	Serum	1H-NMR	DAS28 (6.84)	Yes (no TNFi)	**D**, M	↑	3-hydroxyisobutyrate, acetate, NAC, acetoacetate, acetone
						↓	valine, isoleucine, lactate, alanine, creatinine, GPC APC, histidine
Zhou 2016 [49]	Serum	GC–MS	N/A	N/A	**D**	↑	fatty acids, cholesterol, carbohydrates
						↓	amino acids, glucose, 1,5-anhydrosorbitol, urate, 2-ketoisocaproate
Li 2018 [54]	Serum	UPLC–HRMS	DAS28 (median 6.40)	N/A	**D**	↑	4-methoxyphenylacetic acid, glutamic acid, leucine, phenylalanine, tryptophan, proline, glyceraldehyde, fumaric acid, cholesterol
						↓	capric acid, argininosuccinic acid, bilirubin
Dubey 2019 [30]	Serum	1H-NMR	N/A	No DMARDs	**D**	↓	compared to reactive arthritis: leucine, valine, phenylalanine, arginine/lysine
Souto-Carneiro 2020 [61]	Serum	1H-single-pulse NMR and CPMG NMR	DAS28-CRP (2.3)	Yes	**D** (RA/PsA)	↑	phenylalanine
						↓	alanine, threonine, leucine, valine, acetate, creatine, lactate, choline, lipid ratios
Luan 2021 [35]	Serum	LC–HRMS	Mixed: low, moderate, high	N/A	**D**	↑	CAR 20:3, aspartyl-phenylalanine, pipecolic acid, PE (18:1), LPE (20:3)
						↓	histidine, phosphatidic acid (28:0)
Koh 2022 [59]	Serum, SF	UPLC–Q-TOF-MS	Mixed: low, high	No lipid modulators	**D**, M	↑	LPC, PC, EtherPC, PE, SM subclasses
Liao 2013 [52]	Blood	N/A	N/A	Yes	**D**	↓	total cholesterol, LDL
Yang 2015 [45]	SF	GC–TOF-MS	Mixed: DAS28 inactive (<3.2), active (>3.2)	N/A	**D**	↑	lactic acid, carnitine, pipecolinic acid, diglycerol, beta-mannosylglycerate
						↓	valine, citric acid, gluconic lactone, glucose, G-1-P, mannose, ribitol, 5-methoxytryptamine
Alonso 2016 [41]	Urine	1H-NMR	Mixed: DAS28 very low (1.7), very high (5.5)	Yes	**D**, M	↑	tyrosine
						↓	N-acetyl amino acids, citrate, alanine, carnitine
Sasaki 2019 [32]	Urine	CE–Q-TOF-MS	Mixed: DAS28-ESR active (>3.2), inactive (<3.2)	No biologicals	**D**, M	↑	1-methyl-4-phenyl-1,2,3,6-tetrahydropyridine, 2-quinolinecarboxylic acid, gibberellic acid, hypotaurine, N-acetylglucosamine 1-phosphate, riboflavin
Hur 2021 [62]	Plasma	UPLC–MS	Mixed: DAS28-CRP (3.0)	Yes	**M**, D	↑	glucuronate
						↓	6-bromotryptophan, bilirubin, biliverdin, N-acetyltryptophan, N-acetyltyrosine, serine, trigonelline
Priori 2015 [39]	Serum	1H-NMR	DAS28-CRP responders (median 4.56), non-res. (median 4.65)	Yes (etanercept plus DMARDs and/or GC)	**Pre**	↑	isoleucine, leucine, valine, alanine, glutamine, tyrosine, glucose
						↓	3-hydroxybutyrate
Cuppen 2016 [63]	Serum	LC–MS	DAS28 (4.5)	No biologicals	**Pre**, M	↑	in good responders: sn1-LPC (15:0), lysine
						↓	in good responders: sn1-LPC (18:3-ω3/ω6), ethanolamine
Takahashi 2019 [40]	Serum	CE–TOF-MS	Mixed: moderate, high	Yes (TNFi or abatacept)	**Pre**	↑	TNFi responders: betonicine; ABT responders: citric acid, quinic acid
						↓	TNFi res.: glycerol 3-phosphate, N-acetylalanine, hexanoic acid, taurine; ABT res.: 3-aminobutyric acid
Kapoor 2013 [42]	Urine	1D-NMR	Severe	Yes	**Pre**	↑	histamine, glutamine, xanthurenic acid
						↓	ethanolamine
Surowiec 2016 [64]	Plasma	LC–MS	None	No	**Pro**	↑	kynurenine, LPC (16:0), hypoxanthine, LPC (14:0), 3-indolelactic acid, PLs, SM
						↓	oleic acid, β-hydroxypalmitic acid, fatty acids, acylcarnitines
Chu 2020 [47]	Plasma	UHPLC–HRMS	Healthy	No	**Pro**	↓	acylcarnitine, cholesterol ester, polyamine
van Halm 2007 [53]	Serum	ELISA, immunoturbidimetric method	Healthy	N/A	**Pro**	↑	total cholesterol, triglycerides, apo B
						↓	HDLc
Myasoedova 2010 [51]	Blood	N/A	N/A	N/A	**Pro**	↓	total cholesterol, LDL
Jonsson 2001 [58]	Serum	Dry chemistry, ELISA	Early	Yes (mixed)	**Ri**	↑	cholesterol, LDL, LDL/HDL ratio

* D, diagnostic; M, monitoring; Pre, predictive; Pro, prognostic; Re, response; Ri, risk; S, safety.

## 3. Psoriatic Arthritis

Several studies also investigated the metabolic profile of PsA and correlated it with diagnosis and disease activity monitoring; however, the number is considerably smaller compared to the number of studies that deal with the metabolic profile of RA patients [65]. Nonetheless, candidate biomarkers for PsA could be identified (Table 2) and are presented in the following.

For instance, increased levels of glucuronic acid were found in the serum of PsA patients compared to healthy controls. Since glucuronic acid is a building block of glycosaminoglycans (GAGs), it was suggested that this increase is associated with the release of two GAGs, namely hyaluronan and chondroitin sulfate. Hyaluronan might be released from joint destruction due to inflammation, and the increased chondroitin sulfate levels might be caused by changes in lesional skin, since higher chondroitin sulfate levels can also be found in Ps skin samples [66]. Thus, measuring the levels of glucuronic acid in peripheral blood might improve diagnosis and disease activity monitoring in PsA, as well as in Ps. Furthermore, increased levels of trimethylamine N-oxide (TMAO) were detected in PsA serum samples, and a correlation with disease activity in skin and joints was presented. It was suggested that higher activity of the enzyme FMO3, which metabolizes TMA to TMAO, might cause the observed increase, since FMO3 is known to be upregulated in obese patients, and PsA, as well as Ps, is linked to obesity and metabolic syndrome. Although TMAO is a contributing factor to vascular inflammation, its role in PsA inflammation remains unclear [67] and should be further investigated. As in RA, lower urinary concentrations of citric acid were found in PsA patients compared to healthy controls [41]. In addition to their potential application as a diagnostic biomarker, urinary citric acid levels might also prove suitable for disease activity monitoring, as they exhibited an inverse correlation with disease activity [41].

In a previous study, correlations between different disease activity scores and pro-inflammatory eicosanoids (PGE2, HXB3, and 6,15-dk,dh,PGF1a), as well as anti-inflammatory (11-HEPE, 12-HEPE, and 15-HEPE) eicosanoids, were shown. Additionally, lower levels of anti-inflammatory resolvin D1 were detected in PsA patients with high disease activity. It was suggested that these changes in eicosanoid levels might contribute to either the maintenance or the resolution of inflammation, but more research is still required to elucidate the importance of eicosanoids in the development of PsA [68].

Moreover, monitoring the levels of free 4-hydroxynonenal (4-HNE) and 4-HNE adducts in the mononuclear cells of Ps patients might be suitable to predict the conversion from Ps to PsA if combined with the monitoring of other lipids. Higher adduct levels were found in Ps patients, whereas levels of free 4-HNE were increased in patients who develop PsA. Moreover, 4-HNE is a known biomarker of lipid peroxidation and acts as a lipid mediator with an influence on inflammation and immunological responses. The observed differences in free 4-HNE and 4-HNE adduct levels indicate distinct disease mechanisms in PsA and Ps [69]. However, a recent study could not show any significant differences in the serum samples of PsA converters and non-converters. Although the metabolic coverage of this study was limited due to the employment of a SPME protocol, this finding might suggest that the small molecule metabolic differences in blood samples are too subtle to predict PsA development [65].

Ultimately, reliable prognostic biomarkers for the development of PsA in Ps patients are not yet available, even though the diagnosis of PsA before its clinical manifestation would be desirable in order to initiate early treatment and prevent disease progression. In this respect, it was demonstrated that a diagnostic delay of only 6 months might already cause significantly more joint erosions and impaired physical function [70].

**Table 2:** Changed metabolites in psoriatic arthritis (PsA). Biomarker category highlighted in bold: Corresponding metabolites listed in column “Results”.

Reference	Matrix	Instruments	Disease Activity and/or Severity	Patients Treated?	Biomarker Categories *		Results
Kishikawa 2021 [71]	Plasma	CE–TOF-MS LC–TOF-MS	Ps: PASI (1.8)	Yes (mixed)	**D** (PsA/Ps)	↑	tyramine
						↓	mucic acid
Ambrożewicz 2018 [72]	Plasma	UPLC–QTOF-MS and GC–FID	N/A	No (4 weeks before the study)	**D**	↑	lipid peroxidation products (4-hydroxynonenal, isoprostanes, and neuroprostanes), endocannabinoids (AEA and 2-AG)
						↓	phospholipids and free polyunsaturated fatty acids
Looby 2021 [65]	Serum	HPLC–HRMS (SPME)	Mixed: mild, moderate, severe	No (at baseline levels)	**D**, M	↑	long-chain fatty acids (e.g., 3-hydroxytetradecanedioic acid, 3-hydroxydo-decanedioic acid), 1,11-undecanedicarboxylic acid, eicosanoids (pro- or anti-inflammatory; including prostaglandins, leukotrienes, …)
Souto-Carneiro 2020 [61]	Serum	1H-single-pulse NMR and CPMG NMR	DAS28-CRP (2.3)	Yes	**D** (PsA/RA)	↑	alanine, threonine, leucine, valine, acetate, creatine, lactate, choline, L3/L1, L5/L1, L6/L1
						↓	phenylalanine
Armstrong 2014 [66]	Serum	GC–TOF-MS	PASI (13.2)	Yes	**D**	↑	glucuronic acid
Madsen 2011 [34]	Serum	GC–TOF-MS and UPLC–MS	N/A	Yes (mixed)	**D** (PsA/RA)	↑	glutamine, heptanoic acid, pseudouridine, inosine, guanosine, arabitol, cystine, cysteine, phosphoric acid, succinic acid
						↓	glutamic acid, histidine, cholesterol, threonic acid, aspartic acid, glutamic acid, 1-monooleoylglycerol, arachidonic acid, serine
Coras 2019 [67]	Serum	LC–MS	Mixed: DAS28-PCR (2.74)	Yes (mixed)	**M**	↑	TMAO
Coras 2019 [68]	Serum	UPLC–MS	Mixed: DAS28-CPR high and low (2.72)	Yes	**M**	↑	pro-inflammatory eicosanoids (PGE2, HXB3, 6,15-dk,dh,PGF1a), anti-inflammatory eicosanoids (11-HEPE, 12-HEPE, 15-HEPE)
						↓	anti-inflammatory eicosanoids (8,9-diHETrE, 11,12-diHETrE, 14,15-diHETrE, 19,20-diHDPA, 7,17 DHDPA, resolvin D1, 17-HdoHE)
Alonso 2016 [41]	Urine	1H-NMR	Mixed: DAS28-CPR high and low (2.72)	Yes	**D**, M	↓	N-acetyl amino acids, citrate, alanine, trigonelline, methylsuccinate, carnitine
Kapoor 2013 [42]	Urine	1D-NMR	N/A	Yes	**Pre**	↑	histamine, glutamine, xanthurenic acid
						↓	ethanolamine
Wójcik 2019 [69]	Mononuclear cells	UPLC–TOF-MS	N/A	No (4 weeks before the study)	**D**, Pro	↑	8-isoPGF2α, free 4-HNE, endocannabinoids, eicosanoids (PGE1, LTB4, 13HODE, TXB2)
						↓	eicosanoids (15-d-PGJ2, 15 15-HETE)

* D, diagnostic; M, monitoring; Pre, predictive; Pro, prognostic; Re, response; Ri, risk; S, safety.

## 4. Psoriasis

During our evaluation of the literature, various low-molecular-weight metabolites indicated their potential to be applied as biomarkers for diagnosis, disease activity monitoring, and response prediction in Ps (Table 3) and are therefore discussed below.

In particular, changes in amino acid levels between Ps patients and healthy controls could be determined, e.g., the decrease of glutamine [66] and the increase of ornithine levels [48,73,74] in peripheral blood. In addition, glutamine levels were negatively correlated with the PASI score [31]. Glutamine might be depleted by immune cells and by higher rates of protein biosynthesis. However, the changes in glutamine and ornithine levels might also be explained by the increased keratinocyte hyperproliferation that can be observed in Ps lesional skin [73,74,75]. Additionally, concentrations of free amino acids in peripheral blood were able to predict the treatment response to the TNFi etanercept in a previous study and exhibited a positive correlation with disease severity. Interestingly, it was demonstrated that the metabolic profile of Ps patients was shifted to that of healthy controls after treatment with etanercept [73].

Moreover, differences in concentrations of carnitines and acylcarnitines in peripheral blood were detected between Ps patients and healthy controls, and this might be attributed to increased fatty acid oxidation [31,76] and higher energy consumption of proliferating cells [31,48]. In Ps lesional skin, short-chain acylcarnitines levels were elevated [77]; this elevation might be connected to cachexic conditions that were previously reported for Ps patients [73]. In addition, long-chain acylcarnitines were elevated in Ps lesional skin, and this might be a contributing factor to inflammation and cellular-stress activation [77]. Moreover, perturbations in carnitine and acylcarnitines levels might be caused by the abnormal lipid metabolism that was found in Ps patients. It was discovered that low levels of phosphatidylcholines (PCs) and high levels of lysophosphatidylcholines (LPCs) can be measured in Ps plasma [75,78] and serum [79] samples. Increased degradation of PCs leads to higher concentrations of LPCs that can induce inflammation, generate oxidative stress, and cause atherosclerosis [79]. This is consistent with the observation that psoriasis is associated with obesity and metabolic syndrome. Lipolysis and beta-oxidation were described to be amplified in Ps patients, and this might be caused by the increased production of lipid mediators that are needed to resolve inflammation [76]. The decrease of polyunsaturated fatty acids (PUFAs) in plasma [75] might be linked to the increased production of oxidized derivatives that are associated with inflammation and immune response. In this respect, elevated levels of oxidized linoleic acid and arachidonic acid derivatives were found in the plasma and lesional skin of Ps patients [76]. However, the distribution is different among these two matrices with fast proliferating Ps skin excessively producing pro- and anti-inflammatory lipid mediators, such as 9-HODE and 13-HODE [76]. In the non-lesional skin of Ps patients, no significant metabolic differences were found compared to the skin samples of healthy controls [77]. This finding implies that inflammation processes are probably responsible for metabolic shifts in the skin of Ps patients.

**Table 3:** Changed metabolites in psoriasis (Ps). Biomarker category highlighted in bold: Corresponding metabolites listed in column “Results”.

Reference	Matrix	Instruments	Disease Activity and/or Severity	Patients Treated?	Biomarker Categories *		Results
Chen 2021 [31]	Plasma	UHPLC–qTOF-MS	PASI (10.11)	No	**D**, M	↑	EAAs, BCAAs, carnitines (C6, C18:1-OH)
						↓	glutamine, cysteine, asparagine, carnitines (C16)
Kishikawa 2021 [71]	Plasma	CE–TOF-MS LC–TOF-MS	PASI (1.8)	Yes (mixed)	**D**	↑	ethanolamine phosphate
						↓	nicotinic acid, 20α-hydroxyprogesterone
Li 2019 [75]	Plasma	UPLC–Q-TOF-MS	PASI (9.93)	No	**D**	↑	threonine, leucine, phenylalanine, tryptophan, palmitamide, linoleic amide, oleamide, stearamide, cis-11- eicosenamide, trans-13-docosenamide, uric acid, LysoPCs
						↓	oleic acid, arachidonic acid, N-linoleoyl taurine
Sorokin 2018 [76]	Plasma	LC–MS	N/A	No	**D**	↑	9-,13-HODE, laurylcarnitine, glycerol, adenosine 5′-diphosphate, 7-beta-hydroxycholesterol, xanthosine, N-stearoyltaurine, serotonine
						↓	5-oxoproline, gamma-glutamylglutamine, methionine, cysteine, taurodeoxychoalte
Kamleh 2015 [73]	Plasma	UHPLC–HRMS	Mixed: PASI mild (1.4), severe (16.5)	Yes (mixed)	**D**, M	↑	several proteinogenic AA, citrulline, ornithine, hydroxyproline, cystine, taurine, cytidine, acetylglucosamine, GluCer (C16:0), S1P
						↓	cystathionine
Li 2020 [79]	Serum	UHPLC–qTOF-MS	Mixed: PASI moderate to severe (>10)	N/A	**D**	↑	PAF, LPCs, PI (18:0/16:2)
						↓	cholestane-3,7,12,25-tetrol-3-glucuronide, PCs, LacCer (d18:1/12:0), phenylalanylphenylalanine
Ottas 2017 [48]	Serum	HPLC–MS (biocrates)	Mixed: PASI (1–34)	No	**D**, M	↑	urea, Glu, ornithine, Phe, methioninesulfoxide, several PCs, phytol, taurine, phytol, 1,11-undecanedicarboxylic acid, PE (20:4/0:0)
						↓	acylcarnitines (C9, C7 DC, C12, C10.2, multiple ratios)
Kang 2017 [74]	Serum	GC–MS	PASI (11.4)	No (4 weeks before the study)	**D**, M	↑	several proteinogenic AA, lactic acid, urea
						↓	crotonic acid, azelaic acid, ethanolamine, cholesterol
Armstrong 2014 [66]	Serum	GC–TOF-MS	PASI (9.7)	Yes	**D**	↑	α-ketoglutaric acid
						↓	asparagine, glutamine
Pohla 2020 [77]	Skin	HPLC–MS (biocrates)	N/A	N/A	**D**	↑	AA, acylcarnitines, biogenic amines, LPCs, PCs, histamine, ADMA (Ps lesional skin)
						↓	2 metabolite ratios—citrulline to ornithine and ornithine to arginine (Ps lesional skin)
Sorokin 2018 [76]	Skin	LC–MS	N/A	No	**D**	↑	arachidonic acid metabolites (such as 8-, 12-, 15-hydroxyeicosatetraenoic acid), linoleic acid-derived lipid mediators, 13-hydroxyoctadecadienoic acid

* D, diagnostic; M, monitoring; Pre, predictive; Pro, prognostic; Re, response; Ri, risk; S, safety.

## 5. The Differences

RA, PsA, and Ps represent indications with distinct, although partly overlapping, disease phenotypes. Therefore, it seems reasonable to assume that these individual differences are likewise reflected in their metabolic profiles. In this section, we want to take a closer look at the discriminative characteristics (“the differences”) that previous studies identified in the metabolic signatures of RA, PsA, Ps, and other IMIDs (Table 4). In order to ensure that the observed differences were determined under the same experimental conditions, we considered only the studies that performed direct comparisons between two or more IMIDs. No further analysis was performed.

In a recent study, higher plasma levels of multiple nucleotides, namely ATP, GDP, and ADP, as well as ethanolamine phosphate, 6-aminohexanoic acid, and taurine, were detected in RA patients compared to healthy controls, whereas only xanthine exhibited reduced concentrations in RA patients. Conversely, no significant differences were found in PsA and SLE case-control studies, indicating that these metabolites might probably be utilized as RA-specific diagnostic biomarkers [60]. Another study compared the RA and PsA serum metabolomes and demonstrated varying concentrations of a series of amino acids, organic acids, nucleosides, carbohydrates, lipids (i.e., glycerolipids, cholesterol, and fatty acids), and phosphoric acid [34]. Moreover, the serum metabolomes of seronegative RA and PsA displayed distinct characteristics, as well; that is to say, several amino acids, creatine, acetic acid, lactic acid, and choline showed lower levels in seronegative RA. The only exception was phenylalanine, which was more abundant in RA samples [61]. Additionally, it has been stated before that RA and PsA patients show individual urinary metabolic profiles after treatment with the TNFi infliximab and etanercept, and this might indicate distinct mechanisms of action that could be used for treatment-response prediction [42,80]. In summary, metabolic differences between RA and PsA mainly involve the following classes: nucleotides or nucleotide-related metabolites, amino acids, and lipids [80]. It was previously pointed out that RA, compared to other inflammatory arthritic conditions, features peculiar characteristics regarding the processes linked to HIF (hypoxia-inducible factor), as well as angiogenesis, and this might be an explanation for the observed differences [60].

Taking a closer look at metabolic differences between PsA and Ps, a pilot study revealed that the serum samples of PsA patients had elevated levels of lignoceric acid and decreased levels of α-ketoglutaric acid compared to the samples of Ps patients [66]. Another work reported higher levels of tyramine and lower levels of mucic acid in PsA plasma samples as compared to the samples of Ps patients [71]. In the case of α-ketoglutaric acid, increased collagen synthesis in PsA might explain the observed differences [66]. However, the other findings indicate a strong association between PsA and metabolic syndrome (which is often found in both patient groups, but with a higher prevalence in PsA patients) and perturbed lipid metabolism [66,71]. This is consistent with the previous note that lipid profiles have a major influence on PsA disease activity [65]. Moreover, levels of long-chain fatty acids and various eicosanoid classes exhibited higher concentrations in PsA serum samples compared to Ps [65]. This matches the finding that plasma concentrations of PUFAs, which are used for the production of eicosanoids, were decreased in PsA patients compared to Ps patients [72]. Lipid peroxidation pathways also appear to be dysregulated, since increased serum levels of 1,11-undecanedicarboxylic acid, a metabolite that is linked to peroxisomal disorders, were found in PsA and Ps patients [65]. However, levels of 4-HNE, which is another lipid peroxidation marker and lipid mediator, were higher in PsA patients, and 4-HNE products were more abundant in Ps patients in samples containing mononuclear cells [69]. Both indications, PsA and Ps, show an imbalance in lipid metabolism; however, perturbations in PsA appear to be more substantial. This might also explain why metabolic syndrome is more prevalent in PsA patients compared to Ps patients.

One evaluated study also compared the serum metabolic profiles of RA and SLE patients with the profile of healthy controls and determined lower formic acid levels in SLE patients. A possible explanation for this decrease is an upregulation of the purine nucleotide biosynthesis due to chronic inflammation [29,81]. Although the observed change was not shown for RA patients, another study demonstrated perturbations in purine and pyrimidine concentrations in a RA mouse model [82]. This is also consistent with the aforementioned finding that levels of purine nucleotides are elevated in the plasma samples of RA patients [60].

## 6. The Similarities

**Figure 5:**
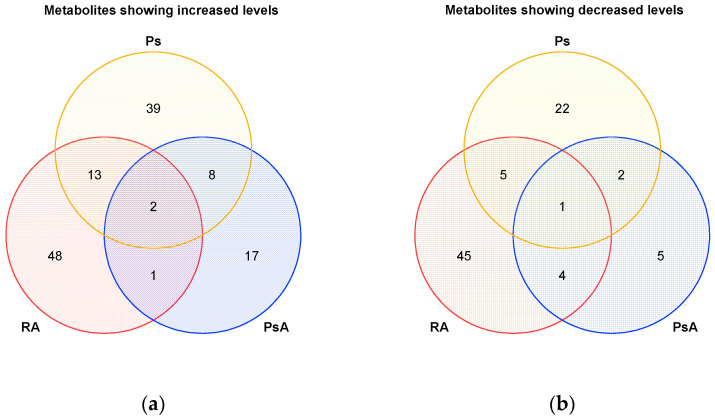
Venn diagrams illustrating potential diagnostic metabolites with (**a**) increased and (**b**) decreased levels for RA, PsA, and Ps. A total of 128 unique metabolites showed increased levels in various matrices, whereas 84 unique metabolites displayed decreased concentrations. A list of the respective metabolites can be found in Supplementary Tables S4 and S5.

IMIDs have similar inflammation processes [83], and metabolic overlaps in autoimmune diseases, such as RA and myasthenia gravis, have already been shown [84]. In fact, we were able to identify metabolites and pathways during our search of the literature that showed similar alterations in RA, PsA, Ps, and other IMIDs (Table 5 and Figure 5). In this context, we summarized potentially diagnostic metabolites in a table, standardized their names via the online resource RefMet, and identified intersections and differences in Microsoft Excel. Corresponding Venn diagrams for RA, PsA, and Ps were created in Origin (Figure 5), and the metabolites indicated in the Venn diagrams are listed in Supplementary Tables S4 and S5. We also included common metabolic changes that were reported in previous works and that were determined from direct comparisons between different patient groups.

For instance, the amino acid metabolism showed similar alterations in all three diseases, and this might generally be explained by an increase in energy demand and the upregulation of protein biosynthesis. Glutamine exhibited lower levels in serum samples of RA and Ps patients, whereas increased concentrations of glutamic acid could be observed in those two diseases [28,30,66]. In addition, the levels of BCAAs, which are known to play a role in oxidative stress, inflammation, and cell migration, were changed in RA and Ps, although to differing degrees. In RA, the levels of BCAAs were found to be decreased [30,37], whereas, in Ps, the levels of BCAAs appeared to be increased [31,75]. In SLE, concentrations of most amino acids, such as alanine, BCAAs, glutamine, and histidine, were reduced in serum samples compared to healthy controls [29]. Likewise, the amino acid metabolism was found to be perturbed in IBD, as serum levels of histidine were lower in Crohn’s disease (CD), as well as in ulcerative colitis (UC) patients [85], and urinary alanine concentrations were diminished in CD patients compared to healthy controls [41].

In the serum samples of RA, PsA [61], Ps [74], IBD [85], and SLE [29] patients, increased lactic acid levels were described, which are likely caused by enhanced glycolytic activity [74,85]. For Ps and PsA, increased aerobic glycolysis was suggested to be advantageous for keratinocyte proliferation [74]. In addition, lactic acid might directly affect inflammation processes, as it was shown to influence the CD4+ T-cell phenotype in arthritis [61]. Nonetheless, significantly different concentrations were found in the serum samples of RA and PsA patients [61], indicating distinct underlying mechanisms associated with lactic acid. However, the evidence regarding lactic acid levels in SLE is conflicting, since concentrations were described to be both higher [29] and lower [86,87,88] in serum samples compared to healthy individuals.

The levels of acylcarnitines and free fatty acids were decreased in peripheral blood of RA [26,64] and Ps [48] patients; this might either be explained by amplified β-oxidation due to the increased energy demand in inflammatory conditions or by decreased lipolysis [64]. Furthermore, increased levels of LPCs were previously described in RA synovial fluid and Ps blood samples [75,79,89]. LPCs are derived from PCs and are building blocks of cellular membranes. They were described to possess pro-inflammatory properties and to be risk factors for other diseases, such as atherosclerosis [75]. Moreover, the COX pathway was found to be involved in the pathogenesis of RA and PsA [68].

**Table 5:** Metabolites and pathways that show similar alterations in multiple IMIDs.

Metabolites or Pathway	Trend	Indication	Matrix	Instruments	Biomarker Categories *	References
Alanine	↓	RA, PsA, Ps, SLE, CD	Urine	1H-NMR	D	Alonso 2016 [41]
	↓	RA, SLE	Serum	1H-NMR	D	Ouyang 2011 [29]
Glutamine	↓	RA	Plasma	GC–Q-TOF-MS	D	He 2021 [28]
	↓	RA	Serum	1H-NMR	D	Dubey 2019 [30]
	↓	Ps	Serum	GC–TOF-MS	D	Armstrong 2014 [66]
	↓	RA, SLE	Serum	1H-NMR	D	Ouyang 2011 [29]
Glutamic acid	↑	RA, Ps	Serum	1H-NMR	D	Dubey 2019 [30]
Histidine	↓	RA, SLE	Serum	1H-NMR	D	Ouyang 2011 [29]
	↓	IBD	Serum	1H-NMR	D	Dawiskiba 2014 [85]
BCAAs	↓	RA	Serum	1H-NMR	D, M	Zabek 2016 [37]
	↓	RA	Serum	1H-NMR	D	Dubey 2019 [30]
	↓	RA, SLE	Serum	1H-NMR	D	Ouyang 2011 [29]
	↑	RA	SF	1H-NMR	D	Dubey 2019 [30]
	↑	Rheumatic conditions	SF	GC–TOF-MS	D	Ahn 2015 [38]
	↑	Ps	Plasma	UHPLC–qTOF-MS	D, M	Chen 2021 [31]
	↑	Ps	Plasma	UPLC–Q-TOF-MS	D	Li 2019 [75]
Ornithine	↑	RA	Plasma	LC–MS	D	Chandrasekharan 2018 [36]
	↑	Ps	Plasma	UHPLC–HRMS	D, M	Kamleh 2015 [73]
	↑	Ps	Serum	HPLC–MS (biocrates)	D	Ottas 2017 [48]
	↑	Ps	Serum	GC–MS	D	Kang 2017 [74]
TCA metabolites	↓	Ps, RA, IBD	Urine	GC–MS	D, Pre	Tsoukalas 2020 [90]
		SLE	Serum	1H-NMR	D	Ouyang 2011 [29]
Hippuric acid	↓	Ps, IBD	Urine	1H-NMR	D	Alonso 2016 [41]
Methylsuccinic acid	↓	PsA, Ps, SLE, IBD	Urine	1H-NMR	D	Alonso 2016 [41]
Citric acid	↓	RA, SLE	Serum	1H-NMR	D	Ouyang 2011 [29]
	↓	RA, PsA, Ps, SLE, IBD	Urine	1H-NMR	D	Alonso 2016 [41]
	↓	PsA, SLE, CD	Urine	1H-NMR	M	Alonso 2016 [41]
Lactic acid	↑	RA	Serum	GC–TOF-MS UPLC–QTOF-MS	D, M	Jiang 2013 [26]
	↑	RA	Serum	1H-NMR	D, M	Young 2013 [43]
	↑	RA, SLE	Serum	1H-NMR	D	Ouyang 2011 [29]
	↑	PsA	Serum	1H-single-pulse NMR and CPMG NMR	D	Souto-Carneiro 2020 [61]
	↑	Ps	Serum	GC–MS	D, M	Kang 2017 [74]
	↑	IBD	Serum	1H-NMR	D	Dawiskiba 2014 [85]
Pyruvic acid	↓	RA, SLE	Serum	1H-NMR	D	Ouyang 2011 [29]
Glucuronic acid	↑	PsA, Ps	Serum	GC–TOF-MS	D	Armstrong 2014 [66]
Trigonelline	↓	PsA, Ps, IBD	Urine	1H-NMR	D	Alonso 2016 [41]
Carnitine	↓	RA, PsA	Urine	1H-NMR	D	Alonso 2016 [41]
Acylcarnitines and free fatty acids	↓	RA	Plasma	LC–MS	Pro	Surowiec 2016 [64]
	↓	RA	Serum	GC–TOF-MS UPLC–QTOF-MS	D, M	Jiang 2013 [26]
	↓	Ps	Serum	HPLC–MS (biocrates)	D, M	Ottas 2017 [48]
1,11-undecanedicarboxylic acid	↑	PsA, Ps	Serum	HPLC–HRMS (SPME)	D, M	Looby 2021 [65]
	↑	Ps	Serum	HPLC–MS (biocrates)	D, M	Ottas 2017 [48]
PC: Phosphocholine	↓	RA, SLE	Serum	1H-NMR	D	Ouyang 2011 [29]
LPCs	↑	RA	SF	UHPLC–qTOF-MS	D	Nieminen 2022 [89]
	↑	Ps	Plasma	UPLC–Q-TOF-MS	D	Li 2019 [75]
	↑	Ps	Serum	UPLC–Q-TOF-MS	D	Li 2020 [79]
COX pathway	↑↓	RA, PsA	Serum	UPLC–MS	M	Coras 2019 [68]

* D, diagnostic; M, monitoring; Pre, predictive; Pro, prognostic; Re, response; Ri, risk; S, safety.

In PsA and Ps, serum levels of glucuronic acid were elevated in both patient groups compared to healthy controls [66], and this might be explained by the association of glucuronic acid with keratinocyte proliferation and epidermal differentiation [64]. As mentioned before, levels of 1,11-undecanedicarboxylic acid, a metabolite that was linked to peroxisomal disorders [65], were found to be elevated in the serum samples of both Ps [48] and PsA [65].

Citric acid has great potential to be employed as a versatile biomarker for several IMIDs, including RA, PsA, Ps, SLE, and IBD [41]. The observed decrease in citric acid levels in urine is probably again the result of increased energy demand due to inflammation [85], since citric acid is a TCA intermediate and is consumed for energy generation. Additionally, another study revealed that further urinary TCA metabolites were decreased in RA and Ps patients, as well as in patients suffering from IBD, and that they could be employed as biomarkers for early diagnosis and disease prediction [90]. Moreover, TCA metabolites were repeatedly described to be decreased in SLE serum samples [29,87,91]. Altered levels of TCA metabolites might result from upregulated energy production in the context of the TCA cycle [29]. Using citric acid and other TCA metabolites as biomarkers offers a major advantage, as urine samples can be collected fast and non-invasively. In this regard, the urine metabolome might be of major importance for diagnosis, disease activity monitoring, and treatment prediction [92] in IMIDs. One study even demonstrated that the same metabolic alterations can be observed in the urine samples of RA and PsA patients after the administration of TNFi, suggesting that drug responses are likely based on a common mechanism in those two diseases [42].

Even more commonalities between multiple IMIDs were previously described. For example, the urinary metabolomes of certain IMIDs were shown to form aggregates in a clustering analysis, i.e., PsA and Ps, CD and UC, and RA and SLE [41]. Interestingly, another study also reported similar metabolic alterations in RA and SLE patients; however, these alterations seemed more prominent in SLE [29]. Besides the aforementioned lower levels of citric acid that were shared across various IMIDs, decreased urinary concentrations of alanine were described in RA, PsA, Ps, SLE, and CD patients [41]. Additionally, lower levels of methylsuccinic acid were found in urine samples from patients that suffer from PsA, Ps, SLE, and IBD, and urinary trigonelline levels seemed to be decreased in PsA, Ps, and IBD patients [41]. Hippuric acid levels were also found to be lower in urine samples of Ps and IBD patients [41]. These findings are also partly in accordance with another work that reported decreased urinary concentrations for citric acid, hippuric acid, and trigonelline in IBD patients [85]. In SLE patients, as well as in RA patients, decreased serum levels of phosphocholine were detected which might be linked to its upregulated consumption to synthesize PCs for cell membranes [29].

Most of the described similarities were determined through the comparison of multiple studies that investigated discriminative metabolites between specific IMID patient groups and healthy controls while using various experimental setups. Nevertheless, these findings could be hinting at common disease mechanisms or mutual dysregulated pathways that might be underlying in the present IMIDs and that might be treated with similar therapies. In order to identify reliable biomarkers, the determination of metabolic differences and similarities via direct comparisons between multiple patient groups under the same experimental conditions need to be performed. Although some previous studies already adopted this approach [29,41,90], more studies that consistently investigate the metabolic profiles and treatment options of patients from different IMIDs are strongly required.

## 7. Conclusions

The application of low-molecular-weight metabolites as biomarkers in precision medicine holds promise for improving diagnosis, disease activity monitoring, and treatment-response prediction in IMIDs such as RA, PsA, and Ps. Nevertheless, there is a lack of reliable biomarkers for these diseases, and their use in a routine clinical setting seems far away, since biomarkers are often nonspecific, and their application is not sufficiently studied or validated. Whereas certain metabolic alterations were only found in one indication, our evaluation of the literature pointed out that others were shared across multiple IMIDs, thus suggesting that similar disease mechanisms might be underlying. In addition, examples were described in which dysregulated pathways were adjusted by shifting the metabolic profile of IMID patients to that one of healthy controls or in which the same metabolic alterations in different patient groups could be observed after particular drug treatments were carried out. We propose that the treatment of specific pathways and, thereby, distinct patient subgroups, i.e., precision medicine, should be favored over the treatment of mere indications. Therefore, studies that integrate omics analyses of large patient cohorts from different IMIDs should be performed to identify and validate common, as well as unique, biomarkers. In this way, metabolic profiles which are characteristic of specific diseases could be identified. Ultimately, pathomechanisms and treatment options of patient subgroups across several IMIDs could be elucidated, and outcomes could be improved.

## Figures and Tables

**Table 4:** Changes in metabolites and pathways that appear to be disease-specific for RA, PsA, or Ps.

Metabolites or Pathway	Trend	Indication	Matrix	Instruments	Biomarker Categories *	References
Nucleotides (ATP, GDP, ADP)	↑	RA (compared to PsA and SLE)	Plasma	CE–TOF-MS	D	Kishikawa 2021 [60]
Ethanolamine phosphate	↑					
6-Aminohexanoic acid	↑					
Taurine	↑					
Xanthine	↓					
Glutamine	↑	RA (compared to PsA)	Serum	GC–TOF-MS UPLC–MS	D	Madsen 2011 [34]
Heptanoic acid	↑					
Succinic acid	↑					
Pseudouridine	↑					
Inosine	↑					
Guanosine	↑					
Arabitol	↑					
Cystine	↑					
Cysteine	↑					
Phosphoric acid	↑					
Aspartic acid	↓					
Glutamic acid	↓					
Histidine	↓					
Serine	↓					
Arachidonic acid	↓					
Cholesterol	↓					
Threonic acid	↓					
1-Monooleoylglycerol	↓					
Alanine, Threonine, Leucine, Valine	↑	PsA (compared to seronegative RA)	Serum	1H-single-pulse NMR and CPMG NMR	D	Souto-Carneiro 2020 [61]
Acetic acid	↑					
Lactic acid	↑					
Choline	↑					
Creatine	↑					
Phenylalanine	↓					
Specific lipid ratios	↓					
Lignoceric acid	↑	PsA (compared to Ps)	Serum	GC–TOF-MS	D	Armstrong 2014 [66]
α-Ketoglutaric acid	↓					
Tyramine	↑	PsA (compared to Ps)	Plasma	CE–TOF-MS LC–TOF-MS	D	Kishikawa 2021 [71]
Mucic acid	↓					
Phospholip. LA (18:2)	↓	PsA (compared to Ps)	Plasma	UPLC–QTOF-MS GC-FID	D	Ambrożewicz 2018 [72]
Phospholip. LA (18:3)	↓					
Free AA (20:4)	↓					
Free DHA (22:6)	↓					
Long-chain fatty acids	↑	PsA (compared to Ps)	Serum	HPLC–HRMS (SPME)	D, M	Looby 2021 [65]
1,11-Undecanedicarboxylic acid	↑					
Eicosanoids	↑					
8-isoPGF2α	↑	PsA (compared to Ps)	Mononuclear cells	UPLC–TOF-MS	D, Pro	Wójcik 2019 [69]
4-HNE	↑					
4-HNE adducts	↓					
15-d-PGJ2	↓					
15-HETE	↓					
Formic acid	↓	SLE (compared to RA)	Serum	1H-NMR	D	Ouyang 2011 [29]

* D, diagnostic; M, monitoring; Pre, predictive; Pro, prognostic; Re, response; Ri, risk; S, safety.

## Data Availability

Not applicable.

## Supplementary Materials

The following supporting information can be downloaded at https://www.mdpi.com/article/10.3390/jpm12060924/s1. Table S1: Literature overview. Table S2: Biomarker categories. Table S3: Search and MeSH terms. Table S4: List of metabolites depicted in Venn diagram Figure 5a. Table S5: List of metabolites depicted in Venn diagram Figure 5b.

## References

[1] Surace A.E.A., Hedrich C.M. (2019). The Role of Epigenetics in Autoimmune/Inflammatory Disease. Front. Immunol..

[2] Van Kempen T.S., Wenink M.H., Leijten E.F.A., Radstake T.R.D.J., Boes M. (2015). Perception of self: Distinguishing autoimmunity from autoinflammation. Nat. Rev. Rheumatol..

[3] McGonagle D., McDermott M.F. (2006). A proposed classification of the immunological diseases. PLoS Med..

[4] Simon D., Watad A., Rodrigues-Manica S., Perricone C. (2021). Editorial: Early Origins of Psoriatic Arthritis. Front. Med..

[5] Gibofsky A. (2012). Overview of Epidemiology, Pathophysiology, and Diagnosis of Rheumatoid Arthritis. Suppl. Featured Publ..

[6] Kalinkovich A., Gabdulina G., Livshits G. (2018). Autoimmunity, inflammation, and dysbiosis mutually govern the transition from the preclinical to the clinical stage of rheumatoid arthritis. Immunol. Res..

[7] Wasserman A.M. (2011). Diagnosis and management of rheumatoid arthritis. Am. Fam. Physician.

[8] Weyand C.M., Goronzy J.J. (2021). The immunology of rheumatoid arthritis. Nat. Immunol..

[9] Arend W.P., Firestein G.S. (2012). Pre-rheumatoid arthritis: Predisposition and transition to clinical synovitis. Nat. Rev. Rheumatol..

[10] Agnihotri P., Monu, Ramani S., Chakraborty D., Saquib M., Biswas S. (2021). Differential Metabolome in Rheumatoid Arthritis: A Brief Perspective. Curr. Rheumatol. Rep..

[11] Ingegnoli F., Castelli R., Gualtierotti R. (2013). Rheumatoid factors: Clinical applications. Dis. Markers.

[12] De Cock D., van der Elst K., Stouten V., Peerboom D., Joly J., Westhovens R., Verschueren P. (2019). The perspective of patients with early rheumatoid arthritis on the journey from symptom onset until referral to a rheumatologist. Rheumatol. Adv. Pract..

[13] McInnes I.B., Buckley C.D., Isaacs J.D. (2016). Cytokines in rheumatoid arthritis-shaping the immunological landscape. Nat. Rev. Rheumatol..

[14] Scher J.U., Ogdie A., Merola J.F., Ritchlin C. (2019). Preventing psoriatic arthritis: Focusing on patients with psoriasis at increased risk of transition. Nat. Rev. Rheumatol..

[15] Ogdie A., Schwartzman S., Husni M.E. (2015). Recognizing and managing comorbidities in psoriatic arthritis. Curr. Opin. Rheumatol..

[16] Ritchlin C.T., Colbert R.A., Gladman D.D. (2017). Psoriatic Arthritis. N. Engl. J. Med..

[17] Damiani G., Bragazzi N.L., Karimkhani Aksut C., Wu D., Alicandro G., McGonagle D., Guo C., Dellavalle R., Grada A., Wong P. (2021). The Global, Regional, and National Burden of Psoriasis: Results and Insights from the Global Burden of Disease 2019 Study. Front. Med..

[18] Armstrong A.W., Read C. (2020). Pathophysiology, Clinical Presentation, and Treatment of Psoriasis: A Review. JAMA.

[19] Pennington S.R., FitzGerald O. (2021). Early Origins of Psoriatic Arthritis: Clinical, Genetic and Molecular Biomarkers of Progression from Psoriasis to Psoriatic Arthritis. Front. Med..

[20] Coates L.C., Helliwell P.S. (2017). Psoriatic arthritis: State of the art review. Clin. Med..

[21] Ghasemi M., Nabipour I., Omrani A., Alipour Z., Assadi M. (2016). Precision medicine and molecular imaging: New targeted approaches toward cancer therapeutic and diagnosis. Am. J. Nucl. Med. Mol. Imaging.

[22] Vargas A.J., Harris C.C. (2016). Biomarker development in the precision medicine era: Lung cancer as a case study. Nat. Rev. Cancer.

[23] FDA-NIH Biomarker Working Group BEST (Biomarkers, EndpointS, and Other Tools) Resource. https://www.ncbi.nlm.nih.gov/books/NBK326791/.

[24] Liu X., Locasale J.W. (2017). Metabolomics: A Primer. Trends Biochem. Sci..

[25] Johnson C.H., Ivanisevic J., Siuzdak G. (2016). Metabolomics: Beyond biomarkers and towards mechanisms. Nat. Rev. Mol. Cell. Biol..

[26] Jiang M., Chen T., Feng H., Zhang Y., Li L., Zhao A., Niu X., Liang F., Wang M., Zhan J. (2013). Serum metabolic signatures of four types of human arthritis. J. Proteome Res..

[27] Yoon N., Jang A.-K., Seo Y., Jung B.H. (2021). Metabolomics in Autoimmune Diseases: Focus on Rheumatoid Arthritis, Systemic Lupus Erythematous, and Multiple Sclerosis. Metabolites.

[28] He Z., Liu Z., Gong L. (2021). Biomarker identification and pathway analysis of rheumatoid arthritis based on metabolomics in combination with ingenuity pathway analysis. Proteomics.

[29] Ouyang X., Dai Y., Wen J.L., Wang L.X. (2011). ¹H NMR-based metabolomic study of metabolic profiling for systemic lupus erythematosus. Lupus.

[30] Dubey D., Kumar S., Chaurasia S., Guleria A., Ahmed S., Singh R., Kumari R., Modi D.R., Misra R., Kumar D. (2019). NMR-Based Serum Metabolomics Revealed Distinctive Metabolic Patterns in Reactive Arthritis Compared with Rheumatoid Arthritis. J. Proteome Res..

[31] Chen C., Hou G., Zeng C., Ren Y., Chen X., Peng C. (2021). Metabolomic profiling reveals amino acid and carnitine alterations as metabolic signatures in psoriasis. Theranostics.

[32] Sasaki C., Hiraishi T., Oku T., Okuma K., Suzumura K., Hashimoto M., Ito H., Aramori I., Hirayama Y. (2019). Metabolomic approach to the exploration of biomarkers associated with disease activity in rheumatoid arthritis. PLoS ONE.

[33] Liu Y., Xie Y. (2021). Metabolomics Approach to the Exploration of Amino Acids Metabolism Changes Associated with Disease Progression in a Rat Model of Adjuvant-Induced Arthritis. J Environ. Pathol. Toxicol. Oncol..

[34] Madsen R.K., Lundstedt T., Gabrielsson J., Sennbro C.-J., Alenius G.-M., Moritz T., Rantapää-Dahlqvist S., Trygg J. (2011). Diagnostic properties of metabolic perturbations in rheumatoid arthritis. Arthritis Res. Ther..

[35] Luan H., Gu W., Li H., Wang Z., Lu L., Ke M., Lu J., Chen W., Lan Z., Xiao Y. (2021). Serum metabolomic and lipidomic profiling identifies diagnostic biomarkers for seropositive and seronegative rheumatoid arthritis patients. J. Transl. Med..

[36] Chandrasekharan U.M., Wang Z., Wu Y., Wilson Tang W.H., Hazen S.L., Wang S., Elaine Husni M. (2018). Elevated levels of plasma symmetric dimethylarginine and increased arginase activity as potential indicators of cardiovascular comorbidity in rheumatoid arthritis. Arthritis Res. Ther..

[37] Zabek A., Swierkot J., Malak A., Zawadzka I., Deja S., Bogunia-Kubik K., Mlynarz P. (2016). Application of _1_H NMR-based serum metabolomic studies for monitoring female patients with rheumatoid arthritis. J. Pharm. Biomed. Anal..

[38] Ahn J.K., Kim S., Kim J., Hwang J., Kim K.H., Cha H.-S. (2015). A Comparative Metabolomic Evaluation of Behcet’s Disease with Arthritis and Seronegative Arthritis Using Synovial Fluid. PLoS ONE.

[39] Priori R., Casadei L., Valerio M., Scrivo R., Valesini G., Manetti C. (2015). ¹H-NMR-Based Metabolomic Study for Identifying Serum Profiles Associated with the Response to Etanercept in Patients with Rheumatoid Arthritis. PLoS ONE.

[40] Takahashi S., Saegusa J., Onishi A., Morinobu A. (2019). Biomarkers identified by serum metabolomic analysis to predict biologic treatment response in rheumatoid arthritis patients. Rheumatology.

[41] Alonso A., Julià A., Vinaixa M., Domènech E., Fernández-Nebro A., Cañete J.D., Ferrándiz C., Tornero J., Gisbert J.P., Nos P. (2016). Urine metabolome profiling of immune-mediated inflammatory diseases. BMC Med..

[42] Kapoor S.R., Filer A., Fitzpatrick M.A., Fisher B.A., Taylor P.C., Buckley C.D., McInnes I.B., Raza K., Young S.P. (2013). Metabolic profiling predicts response to anti-tumor necrosis factor α therapy in patients with rheumatoid arthritis. Arthritis Rheum..

[43] Young S.P., Kapoor S.R., Viant M.R., Byrne J.J., Filer A., Buckley C.D., Kitas G.D., Raza K. (2013). The impact of inflammation on metabolomic profiles in patients with arthritis. Arthritis Rheum..

[44] Lauridsen M.B., Bliddal H., Christensen R., Danneskiold-Samsøe B., Bennett R., Keun H., Lindon J.C., Nicholson J.K., Dorff M.H., Jaroszewski J.W. (2010). _1_H NMR spectroscopy-based interventional metabolic phenotyping: A cohort study of rheumatoid arthritis patients. J. Proteome Res..

[45] Yang X.Y., Di Zheng K., Lin K., Zheng G., Zou H., Wang J.M., Lin Y.Y., Chuka C.M., Ge R.S., Zhai W. (2015). Energy Metabolism Disorder as a Contributing Factor of Rheumatoid Arthritis: A Comparative Proteomic and Metabolomic Study. PLoS ONE.

[46] Krähenbühl S., Willer B., Brühlmann P., Hoppeler H., Stucki G. (1999). Carnitine homeostasis in patients with rheumatoid arthritis. Clin. Chim. Acta.

[47] Chu S.H., Cui J., Sparks J.A., Lu B., Tedeschi S.K., Speyer C.B., Moss L., Feser M.L., Kelmenson L.B., Mewshaw E.A. (2020). Circulating plasma metabolites and risk of rheumatoid arthritis in the Nurses’ Health Study. Rheumatology.

[48] Ottas A., Fishman D., Okas T.-L., Kingo K., Soomets U. (2017). The metabolic analysis of psoriasis identifies the associated metabolites while providing computational models for the monitoring of the disease. Arch. Dermatol. Res..

[49] Zhou J., Chen J., Hu C., Xie Z., Li H., Wei S., Wang D., Wen C., Xu G. (2016). Exploration of the serum metabolite signature in patients with rheumatoid arthritis using gas chromatography-mass spectrometry. J. Pharm. Biomed. Anal..

[50] Rantapää-Dahlqvist S., Wållberg-Jonsson S., Dahlén G. (1991). Lipoprotein (a), lipids, and lipoproteins in patients with rheumatoid arthritis. Ann. Rheum. Dis..

[51] Myasoedova E., Crowson C.S., Kremers H.M., Fitz-Gibbon P.D., Therneau T.M., Gabriel S.E. (2010). Total cholesterol and LDL levels decrease before rheumatoid arthritis. Ann. Rheum. Dis..

[52] Liao K.P., Cai T., Gainer V.S., Cagan A., Murphy S.N., Liu C., Churchill S., Shaw S.Y., Kohane I., Solomon D.H. (2013). Lipid and lipoprotein levels and trend in rheumatoid arthritis compared to the general population. Arthritis Care Res..

[53] Van Halm V.P., Nielen M.M.J., Nurmohamed M.T., van Schaardenburg D., Reesink H.W., Voskuyl A.E., Twisk J.W.R., van de Stadt R.J., de Koning M.H.M.T., Habibuw M.R. (2007). Lipids and inflammation: Serial measurements of the lipid profile of blood donors who later developed rheumatoid arthritis. Ann. Rheum. Dis..

[54] Li J., Che N., Xu L., Zhang Q., Wang Q., Tan W., Zhang M. (2018). LC-MS-based serum metabolomics reveals a distinctive signature in patients with rheumatoid arthritis. Clin. Rheumatol..

[55] Plutzky J., Liao K.P. (2018). Lipids in RA: Is Less Not Necessarily More?. Curr. Rheumatol. Rep..

[56] Robertson J., Porter D., Sattar N., Packard C.J., Caslake M., McInnes I., McCarey D. (2017). Interleukin-6 blockade raises LDL via reduced catabolism rather than via increased synthesis: A cytokine-specific mechanism for cholesterol changes in rheumatoid arthritis. Ann. Rheum. Dis..

[57] Charles-Schoeman C., Fleischmann R., Davignon J., Schwartz H., Turner S.M., Beysen C., Milad M., Hellerstein M.K., Luo Z., Kaplan I.V. (2015). Potential mechanisms leading to the abnormal lipid profile in patients with rheumatoid arthritis versus healthy volunteers and reversal by tofacitinib. Arthritis Rheumatol..

[58] Jonsson S.W., Backman C., Johnson O., Karp K., Lundström E., Sundqvist K.G., Dahlqvist S.R. (2001). Increased prevalence of atherosclerosis in patients with medium term rheumatoid arthritis. J. Rheumatol..

[59] Koh J.H., Yoon S.J., Kim M., Cho S., Lim J., Park Y., Kim H.-S., Kwon S.W., Kim W.-U. (2022). Lipidome profile predictive of disease evolution and activity in rheumatoid arthritis. Exp. Mol. Med..

[60] Kishikawa T., Maeda Y., Nii T., Arase N., Hirata J., Suzuki K., Yamamoto K., Masuda T., Ogawa K., Tsuji S. (2021). Increased levels of plasma nucleotides in patients with rheumatoid arthritis. Int. Immunol..

[61] Souto-Carneiro M., Tóth L., Behnisch R., Urbach K., Klika K.D., Carvalho R.A., Lorenz H.-M. (2020). Differences in the serum metabolome and lipidome identify potential biomarkers for seronegative rheumatoid arthritis versus psoriatic arthritis. Ann. Rheum. Dis..

[62] Hur B., Gupta V.K., Huang H., Wright K.A., Warrington K.J., Taneja V., Davis J.M., Sung J. (2021). Plasma metabolomic profiling in patients with rheumatoid arthritis identifies biochemical features predictive of quantitative disease activity. Arthritis Res. Ther..

[63] Cuppen B.V.J., Fu J., van Wietmarschen H.A., Harms A.C., Koval S., Marijnissen A.C.A., Peeters J.J.W., Bijlsma J.W.J., Tekstra J., van Laar J.M. (2016). Exploring the Inflammatory Metabolomic Profile to Predict Response to TNF-α Inhibitors in Rheumatoid Arthritis. PLoS ONE.

[64] Surowiec I., Ärlestig L., Rantapää-Dahlqvist S., Trygg J. (2016). Metabolite and Lipid Profiling of Biobank Plasma Samples Collected Prior to Onset of Rheumatoid Arthritis. PLoS ONE.

[65] Looby N., Roszkowska A., Reyes-Garcés N., Yu M., Bączek T., Kulasingam V., Pawliszyn J., Chandran V. (2021). Serum metabolic fingerprinting of psoriasis and psoriatic arthritis patients using solid-phase microextraction-liquid chromatography-high-resolution mass spectrometry. Metab. Off. J. Metab. Soc..

[66] Armstrong A.W., Wu J., Johnson M.A., Grapov D., Azizi B., Dhillon J., Fiehn O. (2014). Metabolomics in psoriatic disease: Pilot study reveals metabolite differences in psoriasis and psoriatic arthritis. F1000Research.

[67] Coras R., Kavanaugh A., Boyd T., Huynh D., Lagerborg K.A., Xu Y.-J., Rosenthal S.B., Jain M., Guma M. (2019). Choline metabolite, trimethylamine N-oxide (TMAO), is associated with inflammation in psoriatic arthritis. Clin. Exp. Rheumatol..

[68] Coras R., Kavanaugh A., Boyd T., Huynh Q., Pedersen B., Armando A.M., Dahlberg-Wright S., Marsal S., Jain M., Paravar T. (2019). Pro- and anti-inflammatory eicosanoids in psoriatic arthritis. Metab. Off. J. Metab. Soc..

[69] Wójcik P., Biernacki M., Wroński A., Łuczaj W., Waeg G., Žarković N., Skrzydlewska E. (2019). Altered Lipid Metabolism in Blood Mononuclear Cells of Psoriatic Patients Indicates Differential Changes in Psoriasis Vulgaris and Psoriatic Arthritis. Int. J. Mol. Sci..

[70] Haroon M., Gallagher P., FitzGerald O. (2015). Diagnostic delay of more than 6 months contributes to poor radiographic and functional outcome in psoriatic arthritis. Ann. Rheum. Dis..

[71] Kishikawa T., Arase N., Tsuji S., Maeda Y., Nii T., Hirata J., Suzuki K., Yamamoto K., Masuda T., Ogawa K. (2021). Large-scale plasma-metabolome analysis identifies potential biomarkers of psoriasis and its clinical subtypes. J. Dermatol. Sci..

[72] Ambrożewicz E., Wójcik P., Wroński A., Łuczaj W., Jastrząb A., Žarković N., Skrzydlewska E. (2018). Pathophysiological Alterations of Redox Signaling and Endocannabinoid System in Granulocytes and Plasma of Psoriatic Patients. Cells.

[73] Kamleh M.A., Snowden S.G., Grapov D., Blackburn G.J., Watson D.G., Xu N., Ståhle M., Wheelock C.E. (2015). LC-MS metabolomics of psoriasis patients reveals disease severity-dependent increases in circulating amino acids that are ameliorated by anti-TNFα treatment. J. Proteome Res..

[74] Kang H., Li X., Zhou Q., Quan C., Xue F., Zheng J., Yu Y. (2017). Exploration of candidate biomarkers for human psoriasis based on gas chromatography-mass spectrometry serum metabolomics. Br. J. Dermatol..

[75] Li S.-S., Liu Y., Li H., Wang L.-P., Xue L.-F., Yin G.-S., Wu X.-S. (2019). Identification of psoriasis vulgaris biomarkers in human plasma by non-targeted metabolomics based on UPLC-Q-TOF/MS. Eur. Rev. Med. Pharmacol. Sci..

[76] Sorokin A.V., Domenichiello A.F., Dey A.K., Yuan Z.-X., Goyal A., Rose S.M., Playford M.P., Ramsden C.E., Mehta N.N. (2018). Bioactive Lipid Mediator Profiles in Human Psoriasis Skin and Blood. J. Investig. Dermatol..

[77] Pohla L., Ottas A., Kaldvee B., Abram K., Soomets U., Zilmer M., Reemann P., Jaks V., Kingo K. (2020). Hyperproliferation is the main driver of metabolomic changes in psoriasis lesional skin. Sci. Rep..

[78] Zeng C., Wen B., Hou G., Lei L., Mei Z., Jia X., Chen X., Zhu W., Li J., Kuang Y. (2017). Lipidomics profiling reveals the role of glycerophospholipid metabolism in psoriasis. GigaScience.

[79] Li L., Yao D.-N., Lu Y., Deng J.-W., Wei J.-A., Yan Y.-H., Deng H., Han L., Lu C.-J. (2020). Metabonomics Study on Serum Characteristic Metabolites of Psoriasis Vulgaris Patients with Blood-Stasis Syndrome. Front. Pharmacol..

[80] Koussiouris J., Looby N., Anderson M., Kulasingam V., Chandran V. (2021). Metabolomics Studies in Psoriatic Disease: A Review. Metabolites.

[81] Lin Z.-Y., Xu P.-B., Yan S.-K., Meng H.-B., Yang G.-J., Dai W.-X., Liu X.-R., Li J.-B., Deng X.-M., Zhang W.-D. (2009). A metabonomic approach to early prognostic evaluation of experimental sepsis by _1_H NMR and pattern recognition. NMR Biomed..

[82] Weljie A.M., Dowlatabadi R., Miller B.J., Vogel H.J., Jirik F.R. (2007). An inflammatory arthritis-associated metabolite biomarker pattern revealed by 1H NMR spectroscopy. J. Proteome Res..

[83] Coates L.C., FitzGerald O., Helliwell P.S., Paul C. (2016). Psoriasis, psoriatic arthritis, and rheumatoid arthritis: Is all inflammation the same?. Semin. Arthritis Rheum..

[84] Blackmore D., Li L., Wang N., Maksymowych W., Yacyshyn E., Siddiqi Z.A. (2020). Metabolomic profile overlap in prototypical autoimmune humoral disease: A comparison of myasthenia gravis and rheumatoid arthritis. Metab. Off. J. Metab. Soc..

[85] Dawiskiba T., Deja S., Mulak A., Ząbek A., Jawień E., Pawełka D., Banasik M., Mastalerz-Migas A., Balcerzak W., Kaliszewski K. (2014). Serum and urine metabolomic fingerprinting in diagnostics of inflammatory bowel diseases. World J. Gastroenterol..

[86] Wu T., Xie C., Han J., Ye Y., Weiel J., Li Q., Blanco I., Ahn C., Olsen N., Putterman C. (2012). Metabolic disturbances associated with systemic lupus erythematosus. PLoS ONE.

[87] Bengtsson A.A., Trygg J., Wuttge D.M., Sturfelt G., Theander E., Donten M., Moritz T., Sennbro C.-J., Torell F., Lood C. (2016). Metabolic Profiling of Systemic Lupus Erythematosus and Comparison with Primary Sjögren’s Syndrome and Systemic Sclerosis. PLoS ONE.

[88] Guleria A., Pratap A., Dubey D., Rawat A., Chaurasia S., Sukesh E., Phatak S., Ajmani S., Kumar U., Khetrapal C.L. (2016). NMR based serum metabolomics reveals a distinctive signature in patients with Lupus Nephritis. Sci. Rep..

[89] Nieminen P., Hämäläinen W., Savinainen J., Lehtonen M., Lehtiniemi S., Rinta-Paavola J., Lehenkari P., Kääriäinen T., Joukainen A., Kröger H. (2022). Metabolomics of Synovial Fluid and Infrapatellar Fat Pad in Patients with Osteoarthritis or Rheumatoid Arthritis. Inflammation.

[90] Tsoukalas D., Fragoulakis V., Papakonstantinou E., Antonaki M., Vozikis A., Tsatsakis A., Buga A.M., Mitroi M., Calina D. (2020). Prediction of Autoimmune Diseases by Targeted Metabolomic Assay of Urinary Organic Acids. Metabolites.

[91] Yan B., Huang J., Zhang C., Hu X., Gao M., Shi A., Zha W., Shi L., Huang C., Yang L. (2016). Serum metabolomic profiling in patients with systemic lupus erythematosus by GC/MS. Mod. Rheumatol..

[92] Yan D., Afifi L., Jeon C., Trivedi M., Chang H.W., Lee K., Liao W. (2017). The metabolomics of psoriatic disease. Psoriasis.

